# Syntheses, crystal structures and properties of tetra­kis­(3-methyl­pyridine-κ*N*)bis­(iso­thio­cyanato-κ*N*)manganese(II) and tetra­kis­(3-methyl­pyridine-κ*N*)bis­(iso­thio­cyanato-κ*N*)iron(II)

**DOI:** 10.1107/S2056989022006491

**Published:** 2022-06-30

**Authors:** Magdalena Ceglarska, Christoph Krebs, Christian Näther

**Affiliations:** a Institute of Physics, Jagiellonian University, Lojasiewicza 11, 30-348 Kraków, Poland; b Institute of Inorganic Chemistry, University of Kiel, Max-Eyth.-Str. 2, 24118 Kiel, Germany

**Keywords:** crystal structure, manganese(II)thio­cyanate, 3-methyl­pyridine, IR spectra, thermal properties

## Abstract

The reaction of Mn(NCS)_2_ or Fe(NCS)_2_ with 3-methyl­pyridine (C_6_H_7_N) leads to the formation of two isostructural compounds with compositions [Mn(NCS)_2_(C_6_H_7_N)_4_] (**1**) and [Fe(NCS)_2_(C_6_H_7_N)_4_] (**2**). IR spectroscopic investigations indicate that only terminally coordinated thio­cyanate anions are present. This is confirmed by single-crystal structure analysis, which shows that their crystal structures consist of discrete centrosymmetric complexes, in which the metal cations are octa­hedrally coordinated by two N-bonded thio­cyanate anions and four 3-methyl­pyridine ligands. X-ray powder diffraction (XRPD) proves that pure samples have been obtained. Thermogravimetric measurements show that decomposition starts at about 90°C and that the two coligands are removed in one step for **1** whereas for **2** no clearly resolved steps are visible. XRPD measurements of the residue obtained after the first mass loss of **1** show that a new and unknown crystalline compound has been formed.

## Abstract 2

The crystal structures of the title compounds consist of discrete complexes, in which the metal cations are octa­hedrally coordinated.

## 1. Chemical context

For many years we and others have been inter­ested in the synthesis of coordination compounds based on thio­cyanate anions. In this context, we are especially inter­ested in compounds where paramagnetic metal cations are linked by the anionic ligands into networks, because they can show inter­esting magnetic properties (Mautner *et al.*, 2018 ▸; Rams *et al.*, 2020 ▸; Böhme *et al.*, 2020 ▸). Unfortunately, the synthesis of such compounds is sometimes difficult to achieve, because metal cations such as, for example Mn^II^, Fe^II^, Co^II^ or Ni^II^ are not very chalcophilic and prefer to coordinate only to the terminal thio­cyanate N atom. With mono-coordinating ligands this leads to the formation of discrete complexes instead of the desired networks. In several cases, this problem can be solved by using discrete complexes as precursors that on heating lose their coligands stepwise, which can lead to the desired compounds with bridging coordination (Werner *et al.*, 2015*a* ▸; Suckert *et al.*, 2016 ▸). 
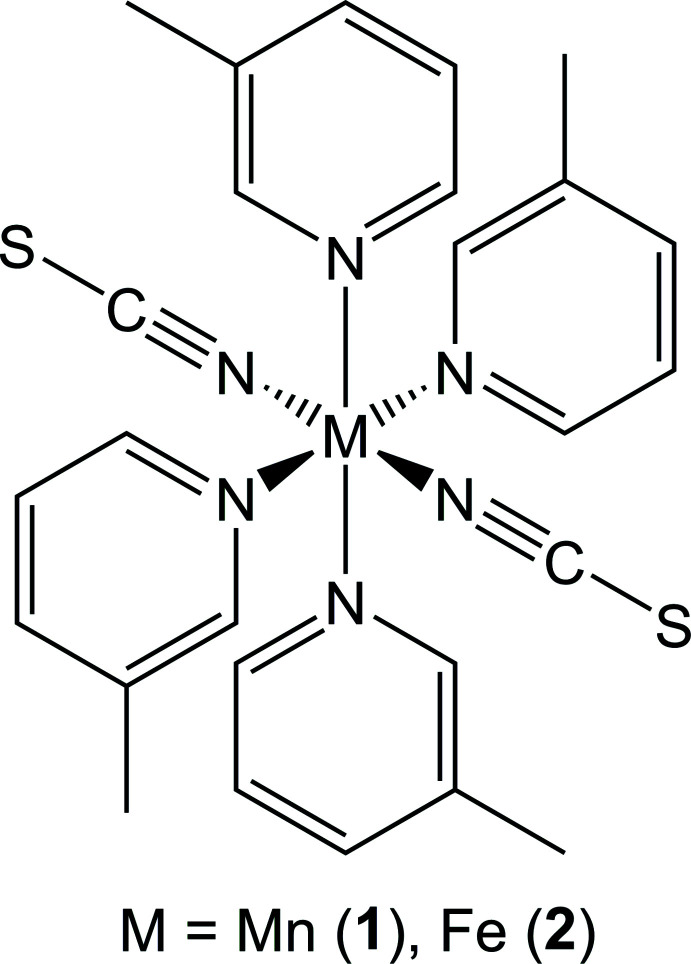


**Figure 1:**
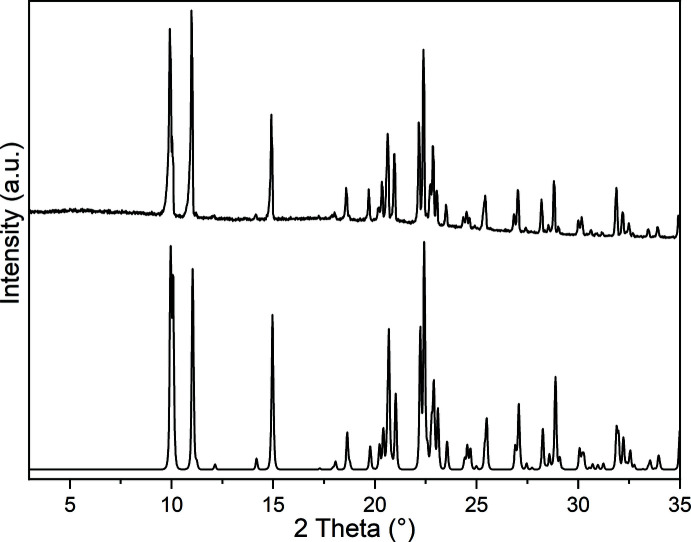
Experimental (top) and calculated (bottom) X-ray powder patterns of compound **1** measured with Cu *K*α radiation.

In the past, many such compounds were prepared following this route, using predominantly pyridine-based ligands that are substituted at the 4-position. In the course of our systematic work, we became inter­ested in 3-methyl­pyridine (3-picoline; C_6_H_7_N) as a coligand. Some compounds have already been reported with this ligand, but bridging coordination of the anionic ligands is observed in only a very few of them (see *Database survey*). This includes compounds with chalcophilic metal cations like Cu^II^, Hg^II^ or Cd^II^ (see *Database survey*). Some time ago we tried to prepare compounds based on cobalt and 3-methyl­pyridine as a coligand, but only octa­hedral discrete complexes were observed (Boeckmann *et al.*, 2011*a* ▸). When the compound Co(NCS)_2_(3-methyl­pyridine)_4_ is investigated by thermogravimetry, the removal of two 3-methyl­pyridine mol­ecules can be detected but, instead of the desired compounds with bridging thio­cyanate anions, only a mononuclear tetra­hedral complex is obtained in which the Co^II^ cations are coordinated by two terminal N-bonded thio­cyanate anions and two 3-methyl­pyridine coligands. With Ni(NCS)_2_, many compounds are known, but all of them consist of discrete complexes with the composition Ni(NCS)_2_(3-methyl­pyridine)_4_ that form channels in which additional solvate mol­ecules are embedded. Two compounds are reported in the Cambridge Structural Database with Mn(NCS)_2_ and Fe(NCS)_2_ and 3-methyl­pyridine as ligand, except for one mixed-metal compound based on manganese and mercury (Małecki, 2017*a* ▸) and therefore, we tried to prepare compounds based on these metal cations. From the reaction of Mn(NCS)_2_ and Fe(NCS)_2_ with 3-methyl­pyridine, two compounds with the composition Mn(NCS)_2_(3-methyl­pyridine)_4_ (**1**) and Fe(NCS)_2_(3-methyl­pyridine)_4_ (**2**) where obtained. IR spectroscopic investigations reveal that the CN stretching vibration of the anionic ligands is observed at 2048 cm^−1^ for **1** and 2046 cm^−1^ for **2**, indicating that only terminal N-bonded thio­cyanate anions are present (Figures S1 and S2 in the supporting information), which was confirmed by structural analysis. Comparison of the experimental X-ray powder diffraction pattern with that calculated from the structure analysis using lattice parameters obtained by measurements performed at room-temperature proves that pure samples have been obtained (Figs. 1 ▸ and 2 ▸). Measurements simultaneously using thermogravimetry and differential thermoanalysis (TG–DTA) reveal that decomposition already starts at about 90°C for both compounds (Figures S3 and S4). Compound **1** shows a mass loss of 34.8%, which is in reasonable agreement with that calculated for the removal of two 3-methyl­pyridine ligands. For compound **2**, a poorly resolved TG curve is observed where the sample mass decreases continuously. The residue of **1** isolated after this mass loss was investigated by XRPD, but the pattern could neither be indexed nor assigned to the possibly isotypic phase Cd(NCS)_2_(3-methyl­pyridine)_2_ (Figure S5; Taniguchi *et al.*, 1987 ▸).

## 2. Structural commentary

**Figure 3:**
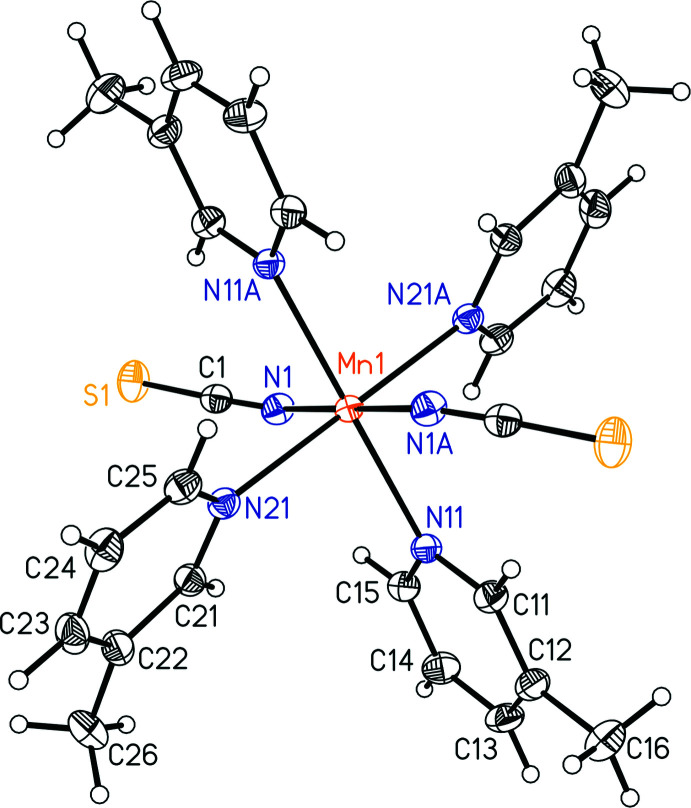
The mol­ecular structure of compound **1** with displacement ellipsoids drawn at the 50% probability level. [Symmetry code: (A) 1 − *x*, 1 − *y*, 1 − *z*.]

Mn(NCS)_2_(3-methyl­pyridine)_4_ (**1**) and Fe(NCS)_2_(3-methyl­pyridine)_4_ (**2**) are isotypic to Co(NCS)_2_(3-methyl­pyridine)_4_ reported in the literature (Boeckmann *et al.*, 2011*a* ▸) and form discrete complexes, in which the metal cations are octa­hedrally coordinated by two terminal N-bonded thio­cyanate anions and two 3-methyl­pyridine coligands (Figs. 3 ▸ and 4 ▸). The asymmetric unit consists of one metal cation that is located on a crystallographic center of inversion as well as one thio­cyanate anion and two 3-methyl­pyridine ligands in general positions. As expected, the *M*—N bond lengths to the negatively charged thio­cyanate anions are shorter than those to the 3-methyl­pyridine coligands and all *M*—N bond lengths are shorter for the Fe compound **2** than for the Mn compound **1** (Tables 1 ▸ and 2 ▸). From the N—*M*—N bonding angles, it is obvious that both octa­hedra are slightly distorted, which can also be seen from the mean octa­hedral quadratic elongation (1.0018 for **1** and 1.0023 for **2**) and the octa­hedral angle variance (1.259°^2^ for **1** and 1.096°^2^ for **2**) calculated by the method of Robinson *et al.* (1971 ▸).

## 3. Supra­molecular features

**Figure 5:**
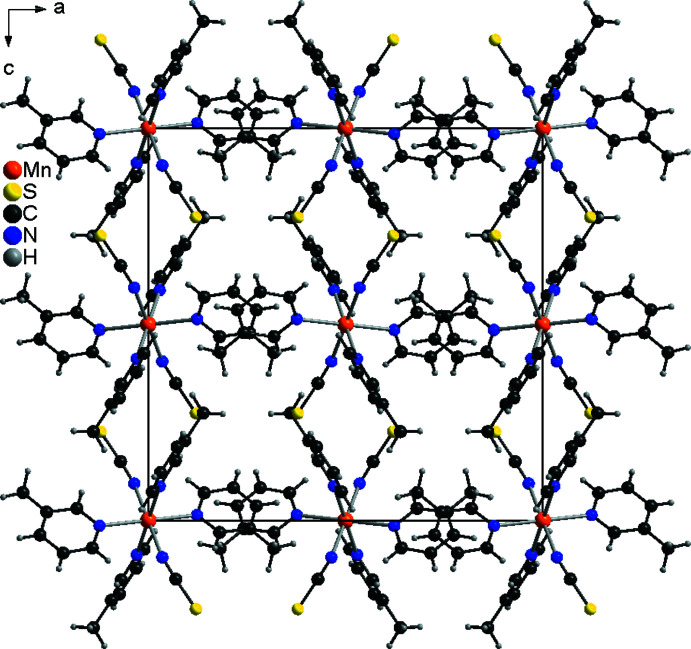
The packing of compound **1** viewed along the crystallographic *b*-axis.

In the extended structures of both compounds, the discrete complexes are arranged into columns that propagate along the crystallographic *b*-axis direction (Fig. 5 ▸). Between these columns, neighboring 3-methyl­pyridine ligands overlap but their ring planes are not parallel, which would be indicative of π–π stacking inter­actions (Fig. 5 ▸). There are some contacts between the C—H hydrogen atoms and the thio­cyanate N and S atoms, but at distances and angles far from those expected for hydrogen bonding (Tables 3 ▸ and 4 ▸).

## 4. Database survey

In the Cambridge Structure Database (CSD, version 5.43, last update November 2021; Groom *et al.*, 2016 ▸) no Fe(NCS)_2_-based compounds with 3-methyl­pyridine as a coligand are reported. With Mn(NCS)_2_ there is only the mixed-metal compound *catena*-[tetra­kis­(thio­cyanato)­bis­(3-methyl­pyri­dine)­manganesemercury] (refcode NAQYOW), in which the Mn^II^ cations are octa­hedrally coordinated by two 3-methyl­pyridine-*N*-oxide ligands and two N-bonding μ-1,3-bridging thio­cyanate anions and are linked to Hg^II^ cations *via* the thio­cyanate S-atoms (Małecki, 2017*a* ▸). The Hg^II^ cations act as tetra­hedral nodes, connecting the Mn^II^ cations into a three-dimensional network.

However, several thio­cyanate compounds with other transition-metal cations and 3-methyl­pyridine as coligand are found in the CSD. With cobalt, three different discrete complexes with the composition Co(NCS)_2_(3-methyl­pyri­dine)_2_(H_2_O)_2_ (EYAREC), Co(NCS)_2_(3-methyl­pyridine)_4_, isotypic to the title compounds (EYAROM and EYAROM01) as well as Co(NCS)_2_(3-methyl­pyridine)_2_ (EYARIG) are reported, in which the Co^II^ cations are octa­hedrally or tetra­hedrally coordinated (Boeckmann *et al.*, 2011*a* ▸; Małecki *et al.*, 2012 ▸). Discrete complexes, in which Ni^II^ cations are octa­hedrally coordinated by two terminal N-bonded thio­cyanate anions and two 3-methyl­pyridine coligands are also known (CIVJEW, CIVJEW10, JICMIR, LAYLAY, LAYLEC, LAYLIG, LAYLOM and LAYLUS) but in their structures cavities are formed, in which additional solvent mol­ecules are embedded (Nassimbeni *et al.*, 1984 ▸, 1986 ▸; Pang *et al.*, 1990 ▸, 1992 ▸). Moreover, one compound with the composition Ni(NCS)_2_(3-methyl­pyridine)_2_(H_2_O)_2_ is also reported (MEGCEH; Tan *et al.*, 2006 ▸).

With Cu^II^, the discrete complexes Cu(NCS)_2_(3-methyl­pyridine)_2_ (ABOTET) and Cu(NCS)_2_(3-methyl­pyridine)_3_ (VEPBAT) with fourfold and fivefold coordinations, respectively, and the chain compound Cu(NCS)(3-methyl­pyridine)_2_ (CUHBEM) are reported (Handy *et al.*, 2017 ▸; Healy *et al.*, 1984 ▸; Kabešová & Kožíšková, 1989 ▸). With Zn(NCS)_2_, the discrete tetra­hedral complex Zn(NCS)_2_(3-methyl­pyridine)_2_ (ETUSAO) is reported (Boeckmann & Näther, 2011*b* ▸), which is isotypic to the corresponding Co(NCS)_2_ compound.

With Cd(NCS)_2_, one compound with the composition Cd(NCS)_2_(3-methyl­pyridine)_2_ (FIYGUP) is observed in which the Cd^II^ cations are linked by pairs of thio­cyanate anions into chains (Taniguchi *et al.*, 1987 ▸). This corresponds exactly to the structural motif in which we are inter­ested and for which many paramagnetic compounds are known with pyridine-based ligands (Werner *et al.*, 2014 ▸, 2015*b* ▸). Finally, two compounds with mercury are also found, *viz. catena*-[tetra­kis­(thio­cyanato)­bis­(3-methyl­pyridine)­manganesemer­cury] (NAQYOW; Małecki, 2017*a* ▸) mentioned above and the isotypic compound where Mn^II^ is replaced by Zn^II^ (QAMSIJ; Małecki, 2017*b* ▸).

## 5. Synthesis and crystallization

**Synthesis**

Ba(SCN)_2_·3H_2_O and 3-picoline were purchased from Alfa Aesar. MnSO_4_·H_2_O was purchased from Merck. A reaction of equimolar amounts of Ba(SCN)_2_·3H_2_O with MnSO_4_·H_2_O in deionized water was performed. After that, the precipitate of BaSO_4_ was filtered off. The filtrate was dried in a rotary evaporator and as a result, a powder of Mn(NCS)_2_ was obtained.

Mn(NCS)_2_(3-methyl­pyridine)_4_: 0.25 mmol of Mn(NCS)_2_ (42.8 mg) were dissolved in 0.5 ml of water and then 1.0 mmol of 3-methyl­pyridine (97.3 µl) were added. The mixture was then heated to 333 K and left at this temperature for 2 d. Afterwards, some colorless crystals were obtained that were suitable for single-crystal X-ray analysis. To obtain powder samples, 0.5 mmol of Mn(NCS)_2_ (85.6 mg) were dissolved in 1.0 ml of ethanol and then 2.0 mmol of 3-methyl­pyridine (194.6 µl) were added. The reaction mixture was stirred for 1 d and the colorless powder was filtered off and dried in the air.

Fe(NCS)_2_(3-methyl­pyridine)_4_: A mixture of 0.25 mmol of FeCl_2_·4H_2_O (49.7 mg) and 0.5 mmol of KSCN (48.6 mg) was dissolved in a mixture of 0.5 ml of water and 0.5 ml of ether. Afterwards, 1.25 mmol of 3-methyl­pyridine (121.6 µl) were added. The mixture was left for 3 d at room temperature, leading to some yellow crystals suitable for single-crystal X-ray diffraction measurements. To obtain powder samples, a mixture of 0.5 mmol of FeCl_2_·4H_2_O (98.6 mg) and 1.0 mmol of KSCN (97.2 mg) was dissolved in 0.5 ml of water. Afterwards, 2.0 mmol of 3-methyl­pyridine (194.6 µl) were added and the reaction mixture was stirred for 1 d. The yellow-colored powder was filtered off and dried in the air.

**Experimental details**

The data collection for single-crystal structure analysis was performed using an XtaLAB Synergy, Dualflex, HyPix diffractometer from Rigaku with Cu *K*α radiation.

The IR spectrum was measured using an ATI Mattson Genesis Series FTIR Spectrometer, control software: *WINFIRST*, from ATI Mattson.

The PXRD measurement was performed with Cu *K*α_1_ radiation (λ = 1.540598 Å) using a Stoe Transmission Powder Diffraction System (STADI P) that is equipped with a MYTHEN 1K detector and a Johansson-type Ge(111) monochromator.

Thermogravimetry and differential thermoanalysis (TG–DTA) measurements were performed in a dynamic nitro­gen atmosphere in Al_2_O_3_ crucibles using a STA-PT 1000 thermobalance from Linseis. The instrument was calibrated using standard reference materials.

## 6. Refinement

Crystal data, data collection and structure refinement details are summarized in Table 5 ▸. The C-bound H atoms were positioned with idealized geometry (methyl H atoms allowed to rotate but not to tip) and were refined isotropically with *U*_iso_(H) = 1.2*U*_eq_(C) (1.5 for methyl H atoms) using a riding model.

## Supplementary Material

Figure S1. IR spectrum of compound 1. The value of the CN-stretching vibration is given. DOI: 10.1107/S2056989022006491/hb8024sup4.png

Figure S2. IR spectrum of compound 2. The value of the CN-stretching vibration is given. DOI: 10.1107/S2056989022006491/hb8024sup5.png

Figure S3. TG-DTA curve of compound 1 measured with 4 degC/min in an nitrogen atmosphere. DOI: 10.1107/S2056989022006491/hb8024sup6.png

Figure S4. TG-DTA curve of compound 2 measured with 4 degC/min in an nitrogen atmosphere. DOI: 10.1107/S2056989022006491/hb8024sup7.png

Figure S5. Experimental XRPD pattern of the product obtained after the first mass loss in a TG measurement of compound 1 (top) and calculated powder pattern of Cd(NCS)2(3-methylpyridine)2 (bottom). DOI: 10.1107/S2056989022006491/hb8024sup8.jpg

Crystal structure: contains datablock(s) 1, 2. DOI: 10.1107/S2056989022006491/hb8024sup1.cif

Structure factors: contains datablock(s) 1. DOI: 10.1107/S2056989022006491/hb80241sup2.hkl

Structure factors: contains datablock(s) 2. DOI: 10.1107/S2056989022006491/hb80242sup3.hkl

CCDC references: 2181394, 2181393

Additional supporting information:  crystallographic information; 3D view; checkCIF report

## Figures and Tables

**Figure 2:**
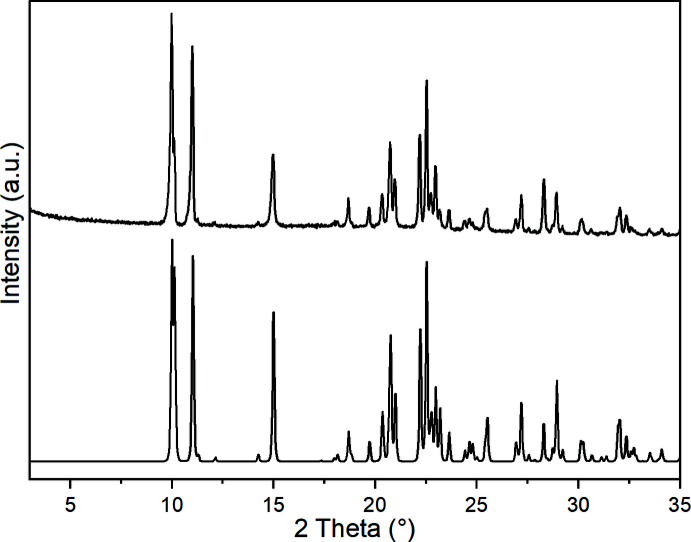
Experimental (top) and calculated (bottom) X-ray powder patterns of compound **2** measured with Cu *K*α radiation.

**Figure 4:**
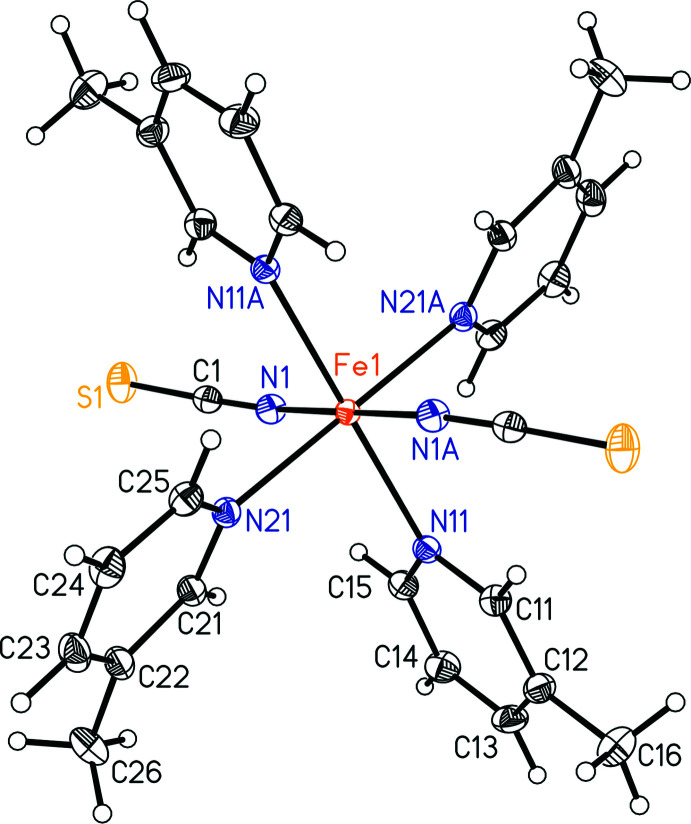
The mol­ecular structure of compound **2** with displacement ellipsoids drawn at the 50% probability level. [Symmetry code: (A) 1 − *x*, 1 − *y*, 1 − *z*.]

**Table 1:** Selected geometric parameters (Å, °) for **1** [Chem scheme1]

Mn1—N1	2.1830 (11)	Mn1—N21	2.2866 (11)
Mn1—N11	2.3306 (11)		
			
N1—Mn1—N11^i^	91.56 (4)	N21—Mn1—N11	89.06 (4)
N1—Mn1—N11	88.44 (4)	N21^i^—Mn1—N11	90.94 (4)
N1^i^—Mn1—N21	90.37 (4)	C1—N1—Mn1	153.96 (10)
N1—Mn1—N21	89.63 (4)		

Symmetry code: (i) 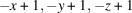 .

**Table 2:** Selected geometric parameters (Å, °) for **2** [Chem scheme1]

Fe1—N1	2.1103 (10)	Fe1—N21	2.2253 (10)
Fe1—N11	2.2779 (10)		
			
N1—Fe1—N11^i^	91.23 (4)	N21—Fe1—N11	89.03 (4)
N1—Fe1—N11	88.77 (4)	N21^i^—Fe1—N11	90.97 (4)
N1^i^—Fe1—N21	90.75 (4)	C1—N1—Fe1	157.12 (10)
N1—Fe1—N21	89.25 (4)		

Symmetry code: (i) 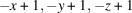 .

**Table 3:** Hydrogen-bond geometry (Å, °) for **1** [Chem scheme1]

*D*—H⋯*A*	*D*—H	H⋯*A*	*D*⋯*A*	*D*—H⋯*A*
C11—H11⋯N1^i^	0.95	2.60	3.2484 (17)	126
C15—H15⋯S1^ii^	0.95	3.00	3.5588 (14)	119
C15—H15⋯N1	0.95	2.52	3.1535 (17)	125

Symmetry codes: (i) 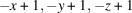 ; (ii) 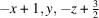 .

**Table 4:** Hydrogen-bond geometry (Å, °) for **2** [Chem scheme1]

*D*—H⋯*A*	*D*—H	H⋯*A*	*D*⋯*A*	*D*—H⋯*A*
C11—H11⋯N1^i^	0.95	2.54	3.1668 (16)	124
C15—H15⋯S1^ii^	0.95	3.00	3.5523 (13)	119
C15—H15⋯N1	0.95	2.48	3.0961 (16)	123

Symmetry codes: (i) 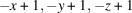 ; (ii) 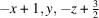 .

**Table 5:** Experimental details

	**1**	**2**
Crystal data		
Chemical formula	[Mn(NCS)_2_(C_6_H_7_N)_4_]	[Fe(NCS)_2_(C_6_H_7_N)_4_]
*M*_r_	543.60	544.51
Crystal system, space group	Orthorhombic, *P* *b* *c* *n*	Orthorhombic, *P* *b* *c* *n*
Temperature (K)	100	100
*a*, *b*, *c* (Å)	17.47811 (10), 8.93570 (6), 17.36177 (10)	17.3733 (1), 8.94119 (5), 17.24862 (10)
*V* (Å^3^)	2711.55 (3)	2679.37 (3)
*Z*	4	4
Radiation type	Cu *K*α	Cu *K*α
μ (mm^−1^)	5.60	6.17
Crystal size (mm)	0.18 × 0.15 × 0.1	0.16 × 0.15 × 0.15

Data collection		
Diffractometer	XtaLAB Synergy, Dualflex, HyPix	XtaLAB Synergy, Dualflex, HyPix
Absorption correction	Multi-scan (*CrysAlis PRO*; Rigaku OD, 2021 ▸)	Multi-scan (*CrysAlis PRO*; Rigaku OD, 2021 ▸)
*T*_min_, *T*_max_	0.786, 1.000	0.555, 1.000
No. of measured, independent and observed [*I* > 2σ(*I*)] reflections	23041, 2918, 2841	22225, 2875, 2804
*R*_int_	0.021	0.020
(sin θ/λ)_max_ (Å^−1^)	0.638	0.638

Refinement		
*R*[*F* ^2^ > 2σ(*F* ^2^)], *wR*(*F* ^2^), *S*	0.028, 0.078, 1.07	0.026, 0.073, 1.06
No. of reflections	2918	2875
No. of parameters	162	163
H-atom treatment	H-atom parameters constrained	H-atom parameters constrained
Δρ_max_, Δρ_min_ (e Å^−3^)	0.45, −0.35	0.39, −0.28

Computer programs: *CrysAlis PRO* (Rigaku OD, 2021 ▸), *SHELXT2014/5* (Sheldrick, 2015*a* ▸), *SHELXL2016/6* (Sheldrick, 2015*b* ▸), *DIAMOND* (Brandenburg & Putz, 1999 ▸) and *publCIF* (Westrip, 2010 ▸).

## supplementary crystallographic information

### Tetrakis(3-methylpyridine-κN)bis(isothiocyanato-κN)manganese(II) (1) Crystal data

**Table:**

[Mn(NCS)_2_(C_6_H_7_N)_4_]	*D*_x_ = 1.332 Mg m^−^^3^
*M**_r_* = 543.60	Cu *K*α radiation, λ = 1.54184 Å
Orthorhombic, *P**b**c**n*	Cell parameters from 12231 reflections
*a* = 17.47811 (10) Å	θ = 5.1–79.2°
*b* = 8.93570 (6) Å	µ = 5.60 mm^−^^1^
*c* = 17.36177 (10) Å	*T* = 100 K
*V* = 2711.55 (3) Å^3^	Block, intense colourless
*Z* = 4	0.18 × 0.15 × 0.1 mm
*F*(000) = 1132	

### Tetrakis(3-methylpyridine-κN)bis(isothiocyanato-κN)manganese(II) (1) Data collection

**Table:**

XtaLAB Synergy, Dualflex, HyPix diffractometer	2918 independent reflections
Radiation source: micro-focus sealed X-ray tube, PhotonJet (Cu) X-ray Source	2841 reflections with *I* > 2σ(*I*)
Mirror monochromator	*R*_int_ = 0.021
Detector resolution: 10.0000 pixels mm^-1^	θ_max_ = 79.8°, θ_min_ = 5.1°
ω scans	*h* = −16→22
Absorption correction: multi-scan (CrysalisPro; Rigaku OD, 2021)	*k* = −10→11
*T*_min_ = 0.786, *T*_max_ = 1.000	*l* = −22→22
23041 measured reflections	

### Tetrakis(3-methylpyridine-κN)bis(isothiocyanato-κN)manganese(II) (1) Refinement

**Table:**

Refinement on *F*^2^	Primary atom site location: dual
Least-squares matrix: full	Hydrogen site location: inferred from neighbouring sites
*R*[*F*^2^ > 2σ(*F*^2^)] = 0.028	H-atom parameters constrained
*wR*(*F*^2^) = 0.078	*w* = 1/[σ^2^(*F*_o_^2^) + (0.0428*P*)^2^ + 1.4595*P*] where *P* = (*F*_o_^2^ + 2*F*_c_^2^)/3
*S* = 1.07	(Δ/σ)_max_ = 0.001
2918 reflections	Δρ_max_ = 0.45 e Å^−^^3^
162 parameters	Δρ_min_ = −0.35 e Å^−^^3^
0 restraints	

### Tetrakis(3-methylpyridine-κN)bis(isothiocyanato-κN)manganese(II) (1) Special details

**Table:**

Geometry. All esds (except the esd in the dihedral angle between two l.s. planes) are estimated using the full covariance matrix. The cell esds are taken into account individually in the estimation of esds in distances, angles and torsion angles; correlations between esds in cell parameters are only used when they are defined by crystal symmetry. An approximate (isotropic) treatment of cell esds is used for estimating esds involving l.s. planes.

### Tetrakis(3-methylpyridine-κN)bis(isothiocyanato-κN)manganese(II) (1) Fractional atomic coordinates and isotropic or equivalent isotropic displacement parameters (Å^2^)

**Table:**

	*x*	*y*	*z*	*U*_iso_*/*U*_eq_	
Mn1	0.500000	0.500000	0.500000	0.01493 (10)	
S1	0.37925 (2)	0.28776 (4)	0.72629 (2)	0.02871 (11)	
C1	0.43007 (7)	0.32513 (14)	0.65000 (8)	0.0197 (3)	
N1	0.46439 (6)	0.35374 (13)	0.59412 (6)	0.0213 (2)	
N11	0.62368 (6)	0.40622 (13)	0.51371 (6)	0.0187 (2)	
C11	0.68099 (7)	0.45625 (15)	0.46930 (8)	0.0211 (3)	
H11	0.668777	0.523569	0.428777	0.025*	
C12	0.75732 (8)	0.41561 (15)	0.47890 (8)	0.0230 (3)	
C13	0.77419 (8)	0.31480 (16)	0.53720 (9)	0.0260 (3)	
H13	0.825438	0.283210	0.545420	0.031*	
C14	0.71595 (8)	0.26057 (16)	0.58327 (8)	0.0262 (3)	
H14	0.726560	0.191037	0.623264	0.031*	
C15	0.64179 (8)	0.30937 (15)	0.57014 (8)	0.0210 (3)	
H15	0.602055	0.272809	0.602382	0.025*	
C16	0.81796 (9)	0.48323 (18)	0.42866 (10)	0.0328 (3)	
H16A	0.805388	0.465065	0.374453	0.049*	
H16B	0.867527	0.437548	0.440654	0.049*	
H16C	0.820602	0.591251	0.438045	0.049*	
N21	0.52897 (6)	0.68274 (12)	0.58746 (6)	0.0188 (2)	
C21	0.56837 (7)	0.65317 (14)	0.65216 (7)	0.0198 (3)	
H21	0.579930	0.551668	0.663532	0.024*	
C22	0.59328 (8)	0.76267 (16)	0.70361 (8)	0.0228 (3)	
C23	0.57305 (8)	0.91022 (16)	0.68741 (8)	0.0264 (3)	
H23	0.588277	0.988617	0.721038	0.032*	
C24	0.53065 (8)	0.94200 (15)	0.62205 (9)	0.0260 (3)	
H24	0.515523	1.041791	0.610919	0.031*	
C25	0.51069 (8)	0.82605 (15)	0.57323 (8)	0.0220 (3)	
H25	0.482864	0.848780	0.527706	0.026*	
C26	0.63924 (10)	0.72132 (18)	0.77362 (9)	0.0320 (3)	
H26A	0.688861	0.772265	0.771841	0.048*	
H26B	0.647277	0.612794	0.774556	0.048*	
H26C	0.611546	0.751935	0.820100	0.048*	

### Tetrakis(3-methylpyridine-κN)bis(isothiocyanato-κN)manganese(II) (1) Atomic displacement parameters (Å^2^)

**Table:**

	*U*^11^	*U*^22^	*U*^33^	*U*^12^	*U*^13^	*U*^23^
Mn1	0.01338 (16)	0.01517 (16)	0.01624 (17)	−0.00031 (10)	−0.00030 (9)	0.00049 (9)
S1	0.0353 (2)	0.02628 (19)	0.02457 (18)	−0.00033 (14)	0.00961 (14)	0.00401 (13)
C1	0.0195 (6)	0.0158 (6)	0.0238 (6)	0.0011 (5)	−0.0029 (5)	0.0002 (5)
N1	0.0202 (5)	0.0216 (5)	0.0220 (5)	−0.0006 (4)	0.0010 (4)	0.0034 (4)
N11	0.0166 (5)	0.0185 (5)	0.0209 (5)	0.0003 (4)	−0.0011 (4)	−0.0022 (4)
C11	0.0194 (6)	0.0201 (6)	0.0238 (6)	0.0004 (5)	0.0000 (5)	0.0003 (5)
C12	0.0177 (6)	0.0220 (6)	0.0292 (6)	−0.0003 (5)	0.0015 (5)	−0.0028 (5)
C13	0.0166 (6)	0.0272 (7)	0.0341 (8)	0.0029 (5)	−0.0045 (5)	−0.0007 (6)
C14	0.0235 (7)	0.0268 (7)	0.0282 (7)	0.0034 (5)	−0.0050 (5)	0.0044 (6)
C15	0.0203 (6)	0.0205 (6)	0.0222 (6)	−0.0002 (5)	−0.0016 (5)	0.0000 (5)
C16	0.0231 (7)	0.0369 (8)	0.0384 (9)	−0.0003 (6)	0.0074 (6)	0.0014 (6)
N21	0.0168 (5)	0.0180 (5)	0.0217 (5)	−0.0006 (4)	0.0008 (4)	−0.0008 (4)
C21	0.0199 (6)	0.0190 (6)	0.0206 (6)	−0.0009 (5)	0.0006 (5)	−0.0012 (5)
C22	0.0241 (6)	0.0235 (6)	0.0209 (6)	−0.0037 (5)	0.0024 (5)	−0.0031 (5)
C23	0.0296 (7)	0.0215 (6)	0.0279 (7)	−0.0059 (5)	0.0049 (5)	−0.0073 (5)
C24	0.0272 (7)	0.0163 (6)	0.0343 (7)	−0.0002 (5)	0.0054 (6)	−0.0008 (5)
C25	0.0196 (6)	0.0198 (6)	0.0266 (7)	0.0007 (5)	0.0008 (5)	0.0018 (5)
C26	0.0387 (8)	0.0329 (8)	0.0243 (7)	−0.0053 (7)	−0.0066 (6)	−0.0055 (6)

### Tetrakis(3-methylpyridine-κN)bis(isothiocyanato-κN)manganese(II) (1) Geometric parameters (Å, º)

**Table:**

Mn1—N1^i^	2.1830 (11)	C15—H15	0.9500
Mn1—N1	2.1830 (11)	C16—H16A	0.9800
Mn1—N11^i^	2.3307 (11)	C16—H16B	0.9800
Mn1—N11	2.3306 (11)	C16—H16C	0.9800
Mn1—N21	2.2866 (11)	N21—C21	1.3439 (17)
Mn1—N21^i^	2.2866 (11)	N21—C25	1.3427 (17)
S1—C1	1.6293 (14)	C21—H21	0.9500
C1—N1	1.1690 (18)	C21—C22	1.3945 (18)
N11—C11	1.3408 (17)	C22—C23	1.394 (2)
N11—C15	1.3450 (17)	C22—C26	1.503 (2)
C11—H11	0.9500	C23—H23	0.9500
C11—C12	1.3926 (18)	C23—C24	1.385 (2)
C12—C13	1.387 (2)	C24—H24	0.9500
C12—C16	1.500 (2)	C24—C25	1.383 (2)
C13—H13	0.9500	C25—H25	0.9500
C13—C14	1.382 (2)	C26—H26A	0.9800
C14—H14	0.9500	C26—H26B	0.9800
C14—C15	1.3864 (19)	C26—H26C	0.9800
			
N1^i^—Mn1—N1	180.0	N11—C15—H15	118.6
N1—Mn1—N11^i^	91.56 (4)	C14—C15—H15	118.6
N1—Mn1—N11	88.44 (4)	C12—C16—H16A	109.5
N1^i^—Mn1—N11^i^	88.44 (4)	C12—C16—H16B	109.5
N1^i^—Mn1—N11	91.56 (4)	C12—C16—H16C	109.5
N1^i^—Mn1—N21	90.37 (4)	H16A—C16—H16B	109.5
N1—Mn1—N21^i^	90.37 (4)	H16A—C16—H16C	109.5
N1^i^—Mn1—N21^i^	89.63 (4)	H16B—C16—H16C	109.5
N1—Mn1—N21	89.63 (4)	C21—N21—Mn1	121.88 (8)
N11—Mn1—N11^i^	180.00 (5)	C25—N21—Mn1	120.41 (9)
N21—Mn1—N11	89.06 (4)	C25—N21—C21	117.59 (11)
N21^i^—Mn1—N11	90.94 (4)	N21—C21—H21	118.1
N21—Mn1—N11^i^	90.94 (4)	N21—C21—C22	123.88 (12)
N21^i^—Mn1—N11^i^	89.06 (4)	C22—C21—H21	118.1
N21—Mn1—N21^i^	180.0	C21—C22—C26	120.82 (13)
N1—C1—S1	177.78 (12)	C23—C22—C21	117.08 (13)
C1—N1—Mn1	153.96 (10)	C23—C22—C26	122.10 (13)
C11—N11—Mn1	120.95 (9)	C22—C23—H23	120.2
C11—N11—C15	117.23 (11)	C24—C23—C22	119.68 (12)
C15—N11—Mn1	121.61 (9)	C24—C23—H23	120.2
N11—C11—H11	118.0	C23—C24—H24	120.5
N11—C11—C12	124.05 (13)	C25—C24—C23	118.92 (13)
C12—C11—H11	118.0	C25—C24—H24	120.5
C11—C12—C16	120.16 (13)	N21—C25—C24	122.79 (13)
C13—C12—C11	117.41 (13)	N21—C25—H25	118.6
C13—C12—C16	122.41 (13)	C24—C25—H25	118.6
C12—C13—H13	120.2	C22—C26—H26A	109.5
C14—C13—C12	119.58 (12)	C22—C26—H26B	109.5
C14—C13—H13	120.2	C22—C26—H26C	109.5
C13—C14—H14	120.6	H26A—C26—H26B	109.5
C13—C14—C15	118.88 (13)	H26A—C26—H26C	109.5
C15—C14—H14	120.6	H26B—C26—H26C	109.5
N11—C15—C14	122.84 (13)		

Symmetry code: (i) −*x*+1, −*y*+1, −*z*+1.

### Tetrakis(3-methylpyridine-κN)bis(isothiocyanato-κN)manganese(II) (1) Hydrogen-bond geometry (Å, º)

**Table:**

*D*—H···*A*	*D*—H	H···*A*	*D*···*A*	*D*—H···*A*
C11—H11···N1^i^	0.95	2.60	3.2484 (17)	126
C15—H15···S1^ii^	0.95	3.00	3.5588 (14)	119
C15—H15···N1	0.95	2.52	3.1535 (17)	125

Symmetry codes: (i) −*x*+1, −*y*+1, −*z*+1; (ii) −*x*+1, *y*, −*z*+3/2.

### Tetrakis(3-methylpyridine-κN)bis(isothiocyanato-κN)iron(II) (2) Crystal data

**Table:**

[Fe(NCS)_2_(C_6_H_7_N)_4_]	*D*_x_ = 1.350 Mg m^−^^3^
*M**_r_* = 544.51	Cu *K*α radiation, λ = 1.54184 Å
Orthorhombic, *P**b**c**n*	Cell parameters from 17643 reflections
*a* = 17.3733 (1) Å	θ = 2.6–79.3°
*b* = 8.94119 (5) Å	µ = 6.17 mm^−^^1^
*c* = 17.24862 (10) Å	*T* = 100 K
*V* = 2679.37 (3) Å^3^	Prism, intense colourless
*Z* = 4	0.16 × 0.15 × 0.15 mm
*F*(000) = 1136	

### Tetrakis(3-methylpyridine-κN)bis(isothiocyanato-κN)iron(II) (2) Data collection

**Table:**

XtaLAB Synergy, Dualflex, HyPix diffractometer	2875 independent reflections
Radiation source: micro-focus sealed X-ray tube, PhotonJet (Cu) X-ray Source	2804 reflections with *I* > 2σ(*I*)
Mirror monochromator	*R*_int_ = 0.020
Detector resolution: 10.0000 pixels mm^-1^	θ_max_ = 79.8°, θ_min_ = 5.1°
ω scans	*h* = −22→22
Absorption correction: multi-scan (CrysalisPro; Rigaku OD, 2021)	*k* = −11→7
*T*_min_ = 0.555, *T*_max_ = 1.000	*l* = −22→20
22225 measured reflections	

### Tetrakis(3-methylpyridine-κN)bis(isothiocyanato-κN)iron(II) (2) Refinement

**Table:**

Refinement on *F*^2^	Hydrogen site location: inferred from neighbouring sites
Least-squares matrix: full	H-atom parameters constrained
*R*[*F*^2^ > 2σ(*F*^2^)] = 0.026	*w* = 1/[σ^2^(*F*_o_^2^) + (0.0401*P*)^2^ + 1.5203*P*] where *P* = (*F*_o_^2^ + 2*F*_c_^2^)/3
*wR*(*F*^2^) = 0.073	(Δ/σ)_max_ = 0.001
*S* = 1.06	Δρ_max_ = 0.39 e Å^−^^3^
2875 reflections	Δρ_min_ = −0.28 e Å^−^^3^
163 parameters	Extinction correction: SHELXL2016/6 (Sheldrick 2015b), Fc^*^=kFc[1+0.001xFc^2^λ^3^/sin(2θ)]^-1/4^
0 restraints	Extinction coefficient: 0.00049 (7)
Primary atom site location: dual	

### Tetrakis(3-methylpyridine-κN)bis(isothiocyanato-κN)iron(II) (2) Special details

**Table:**

Geometry. All esds (except the esd in the dihedral angle between two l.s. planes) are estimated using the full covariance matrix. The cell esds are taken into account individually in the estimation of esds in distances, angles and torsion angles; correlations between esds in cell parameters are only used when they are defined by crystal symmetry. An approximate (isotropic) treatment of cell esds is used for estimating esds involving l.s. planes.

### Tetrakis(3-methylpyridine-κN)bis(isothiocyanato-κN)iron(II) (2) Fractional atomic coordinates and isotropic or equivalent isotropic displacement parameters (Å^2^)

**Table:**

	*x*	*y*	*z*	*U*_iso_*/*U*_eq_	
Fe1	0.500000	0.500000	0.500000	0.01130 (10)	
S1	0.38206 (2)	0.28452 (4)	0.72572 (2)	0.02425 (11)	
C1	0.43143 (7)	0.32807 (13)	0.64874 (7)	0.0156 (2)	
N1	0.46454 (6)	0.36130 (12)	0.59232 (6)	0.0167 (2)	
N11	0.62142 (6)	0.40757 (12)	0.51376 (6)	0.0149 (2)	
C11	0.67967 (7)	0.45813 (14)	0.46972 (7)	0.0177 (2)	
H11	0.667825	0.526986	0.429511	0.021*	
C12	0.75625 (7)	0.41613 (15)	0.47922 (8)	0.0191 (3)	
C13	0.77283 (7)	0.31360 (15)	0.53734 (8)	0.0219 (3)	
H13	0.824265	0.281211	0.545588	0.026*	
C14	0.71377 (8)	0.25904 (15)	0.58315 (8)	0.0220 (3)	
H14	0.724078	0.188658	0.623079	0.026*	
C15	0.63926 (7)	0.30874 (14)	0.56985 (7)	0.0175 (2)	
H15	0.599047	0.271369	0.601743	0.021*	
C16	0.81754 (8)	0.48373 (17)	0.42911 (10)	0.0285 (3)	
H16A	0.806439	0.461886	0.374566	0.043*	
H16B	0.867663	0.441242	0.443056	0.043*	
H16C	0.818606	0.592272	0.436935	0.043*	
N21	0.52872 (6)	0.67812 (12)	0.58534 (6)	0.0151 (2)	
C21	0.56858 (7)	0.64878 (14)	0.65039 (7)	0.0162 (2)	
H21	0.580676	0.547467	0.661651	0.019*	
C22	0.59325 (8)	0.75852 (15)	0.70240 (7)	0.0188 (3)	
C23	0.57244 (8)	0.90588 (15)	0.68632 (8)	0.0222 (3)	
H23	0.587559	0.984287	0.720223	0.027*	
C24	0.52958 (8)	0.93723 (14)	0.62059 (8)	0.0215 (3)	
H24	0.514030	1.036811	0.609476	0.026*	
C25	0.50971 (7)	0.82099 (14)	0.57126 (8)	0.0182 (3)	
H25	0.481440	0.843481	0.525582	0.022*	
C26	0.63958 (9)	0.71736 (17)	0.77282 (8)	0.0274 (3)	
H26A	0.689042	0.769994	0.771510	0.041*	
H26B	0.648640	0.609178	0.773221	0.041*	
H26C	0.611273	0.746064	0.819661	0.041*	

### Tetrakis(3-methylpyridine-κN)bis(isothiocyanato-κN)iron(II) (2) Atomic displacement parameters (Å^2^)

**Table:**

	*U*^11^	*U*^22^	*U*^33^	*U*^12^	*U*^13^	*U*^23^
Fe1	0.01186 (15)	0.01149 (16)	0.01054 (16)	−0.00048 (9)	0.00004 (9)	0.00100 (9)
S1	0.03164 (19)	0.02224 (18)	0.01886 (17)	−0.00132 (13)	0.00886 (13)	0.00403 (12)
C1	0.0169 (6)	0.0121 (5)	0.0178 (6)	0.0006 (4)	−0.0026 (4)	−0.0002 (4)
N1	0.0174 (5)	0.0168 (5)	0.0159 (5)	−0.0005 (4)	0.0002 (4)	0.0032 (4)
N11	0.0149 (5)	0.0147 (5)	0.0151 (5)	0.0006 (4)	−0.0013 (4)	−0.0015 (4)
C11	0.0176 (6)	0.0168 (6)	0.0185 (6)	0.0003 (5)	−0.0004 (5)	0.0002 (5)
C12	0.0157 (6)	0.0190 (6)	0.0227 (6)	−0.0008 (5)	0.0006 (5)	−0.0026 (5)
C13	0.0153 (6)	0.0228 (6)	0.0276 (7)	0.0027 (5)	−0.0050 (5)	−0.0014 (5)
C14	0.0217 (6)	0.0218 (6)	0.0224 (6)	0.0039 (5)	−0.0045 (5)	0.0036 (5)
C15	0.0185 (6)	0.0171 (6)	0.0168 (6)	−0.0001 (5)	−0.0012 (5)	0.0003 (5)
C16	0.0209 (7)	0.0325 (8)	0.0319 (8)	−0.0001 (6)	0.0060 (6)	0.0019 (6)
N21	0.0153 (5)	0.0147 (5)	0.0155 (5)	−0.0007 (4)	0.0012 (4)	−0.0012 (4)
C21	0.0174 (6)	0.0160 (6)	0.0151 (6)	−0.0011 (5)	0.0006 (4)	−0.0010 (4)
C22	0.0209 (6)	0.0194 (6)	0.0161 (6)	−0.0036 (5)	0.0019 (5)	−0.0031 (5)
C23	0.0265 (6)	0.0178 (6)	0.0222 (6)	−0.0051 (5)	0.0041 (5)	−0.0060 (5)
C24	0.0241 (6)	0.0130 (6)	0.0275 (7)	−0.0004 (5)	0.0042 (5)	−0.0008 (5)
C25	0.0174 (6)	0.0166 (6)	0.0205 (6)	0.0008 (5)	0.0008 (5)	0.0013 (5)
C26	0.0352 (8)	0.0273 (8)	0.0198 (7)	−0.0040 (6)	−0.0065 (6)	−0.0044 (5)

### Tetrakis(3-methylpyridine-κN)bis(isothiocyanato-κN)iron(II) (2) Geometric parameters (Å, º)

**Table:**

Fe1—N1^i^	2.1103 (10)	C15—H15	0.9500
Fe1—N1	2.1103 (10)	C16—H16A	0.9800
Fe1—N11^i^	2.2780 (10)	C16—H16B	0.9800
Fe1—N11	2.2779 (10)	C16—H16C	0.9800
Fe1—N21	2.2253 (10)	N21—C21	1.3444 (16)
Fe1—N21^i^	2.2253 (10)	N21—C25	1.3416 (16)
S1—C1	1.6279 (13)	C21—H21	0.9500
C1—N1	1.1688 (17)	C21—C22	1.3968 (17)
N11—C11	1.3436 (16)	C22—C23	1.3942 (19)
N11—C15	1.3464 (16)	C22—C26	1.5030 (19)
C11—H11	0.9500	C23—H23	0.9500
C11—C12	1.3922 (18)	C23—C24	1.385 (2)
C12—C13	1.3887 (19)	C24—H24	0.9500
C12—C16	1.4987 (19)	C24—C25	1.3868 (18)
C13—H13	0.9500	C25—H25	0.9500
C13—C14	1.3838 (19)	C26—H26A	0.9800
C14—H14	0.9500	C26—H26B	0.9800
C14—C15	1.3878 (18)	C26—H26C	0.9800
			
N1^i^—Fe1—N1	180.0	N11—C15—H15	118.5
N1—Fe1—N11^i^	91.23 (4)	C14—C15—H15	118.5
N1—Fe1—N11	88.77 (4)	C12—C16—H16A	109.5
N1^i^—Fe1—N11^i^	88.77 (4)	C12—C16—H16B	109.5
N1^i^—Fe1—N11	91.23 (4)	C12—C16—H16C	109.5
N1^i^—Fe1—N21	90.75 (4)	H16A—C16—H16B	109.5
N1—Fe1—N21^i^	90.75 (4)	H16A—C16—H16C	109.5
N1^i^—Fe1—N21^i^	89.25 (4)	H16B—C16—H16C	109.5
N1—Fe1—N21	89.25 (4)	C21—N21—Fe1	121.87 (8)
N11—Fe1—N11^i^	180.00 (5)	C25—N21—Fe1	120.44 (9)
N21—Fe1—N11	89.03 (4)	C25—N21—C21	117.62 (11)
N21^i^—Fe1—N11	90.97 (4)	N21—C21—H21	118.1
N21—Fe1—N11^i^	90.97 (4)	N21—C21—C22	123.85 (12)
N21^i^—Fe1—N11^i^	89.03 (4)	C22—C21—H21	118.1
N21—Fe1—N21^i^	180.00 (4)	C21—C22—C26	120.76 (12)
N1—C1—S1	177.62 (12)	C23—C22—C21	117.16 (12)
C1—N1—Fe1	157.12 (10)	C23—C22—C26	122.08 (12)
C11—N11—Fe1	121.10 (8)	C22—C23—H23	120.2
C11—N11—C15	116.97 (11)	C24—C23—C22	119.58 (12)
C15—N11—Fe1	121.75 (8)	C24—C23—H23	120.2
N11—C11—H11	117.9	C23—C24—H24	120.5
N11—C11—C12	124.24 (12)	C23—C24—C25	118.97 (12)
C12—C11—H11	117.9	C25—C24—H24	120.5
C11—C12—C16	120.16 (12)	N21—C25—C24	122.77 (12)
C13—C12—C11	117.46 (12)	N21—C25—H25	118.6
C13—C12—C16	122.36 (12)	C24—C25—H25	118.6
C12—C13—H13	120.3	C22—C26—H26A	109.5
C14—C13—C12	119.41 (12)	C22—C26—H26B	109.5
C14—C13—H13	120.3	C22—C26—H26C	109.5
C13—C14—H14	120.5	H26A—C26—H26B	109.5
C13—C14—C15	118.97 (12)	H26A—C26—H26C	109.5
C15—C14—H14	120.5	H26B—C26—H26C	109.5
N11—C15—C14	122.94 (12)		

Symmetry code: (i) −*x*+1, −*y*+1, −*z*+1.

### Tetrakis(3-methylpyridine-κN)bis(isothiocyanato-κN)iron(II) (2) Hydrogen-bond geometry (Å, º)

**Table:**

*D*—H···*A*	*D*—H	H···*A*	*D*···*A*	*D*—H···*A*
C11—H11···N1^i^	0.95	2.54	3.1668 (16)	124
C15—H15···S1^ii^	0.95	3.00	3.5523 (13)	119
C15—H15···N1	0.95	2.48	3.0961 (16)	123

Symmetry codes: (i) −*x*+1, −*y*+1, −*z*+1; (ii) −*x*+1, *y*, −*z*+3/2.
