# Supplementary figures and images for: Syntheses, crystal structures and properties of tetra­kis­(3-methyl­pyridine-κN)bis­(iso­thio­cyanato-κN)manganese(II) and tetra­kis­(3-methyl­pyridine-κN)bis­(iso­thio­cyanato-κN)iron(II)
Source: Acta Crystallogr E Crystallogr Commun. 2022 Jun 30;78(Pt 7):755–60. doi: 10.1107/S2056989022006491 (PMC9260356; doi:10.1107/S2056989022006491)

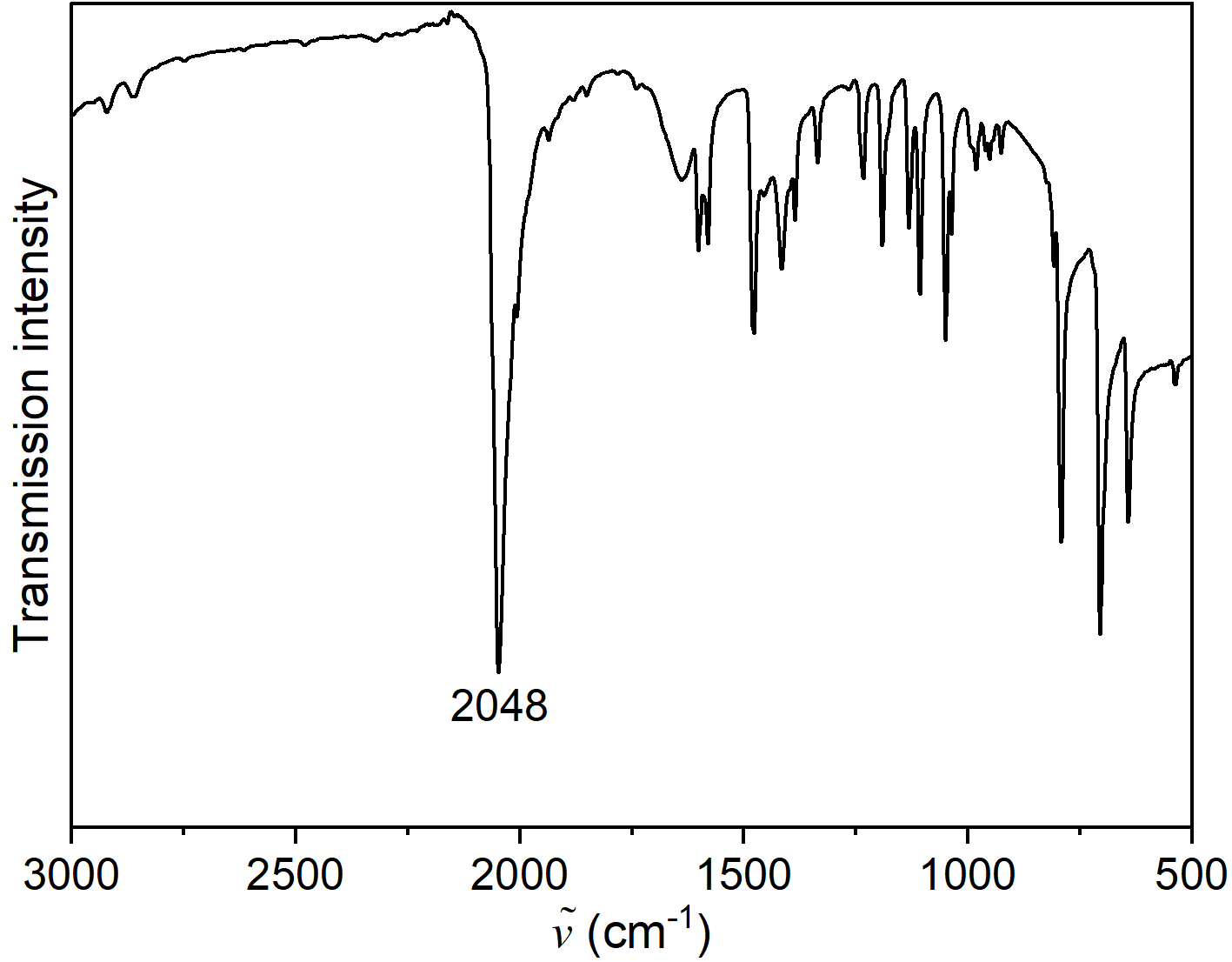

Supplement: Supplementary file 1 [file e-78-00755-sup4.png]

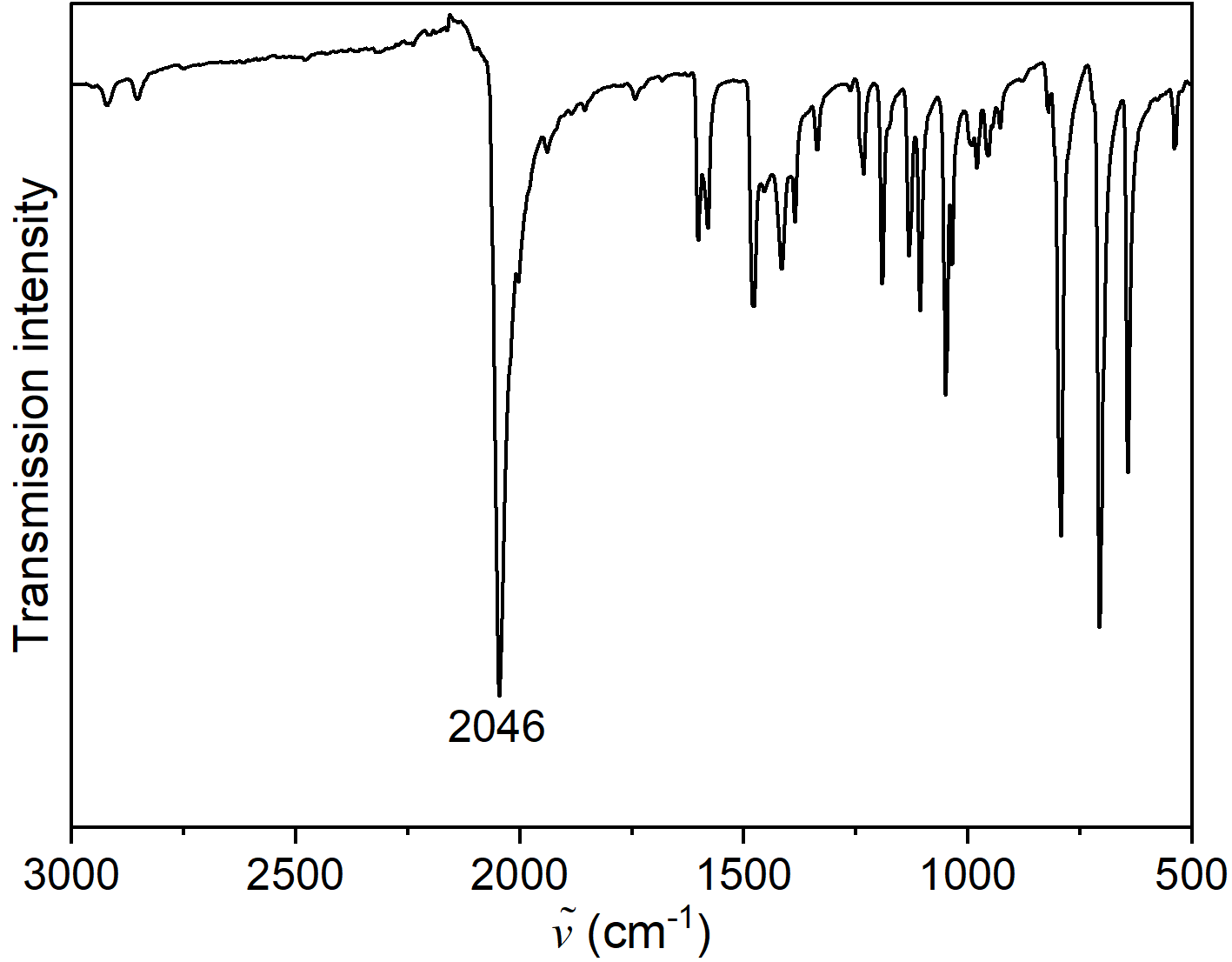

Supplement: Supplementary file 2 [file e-78-00755-sup5.png]

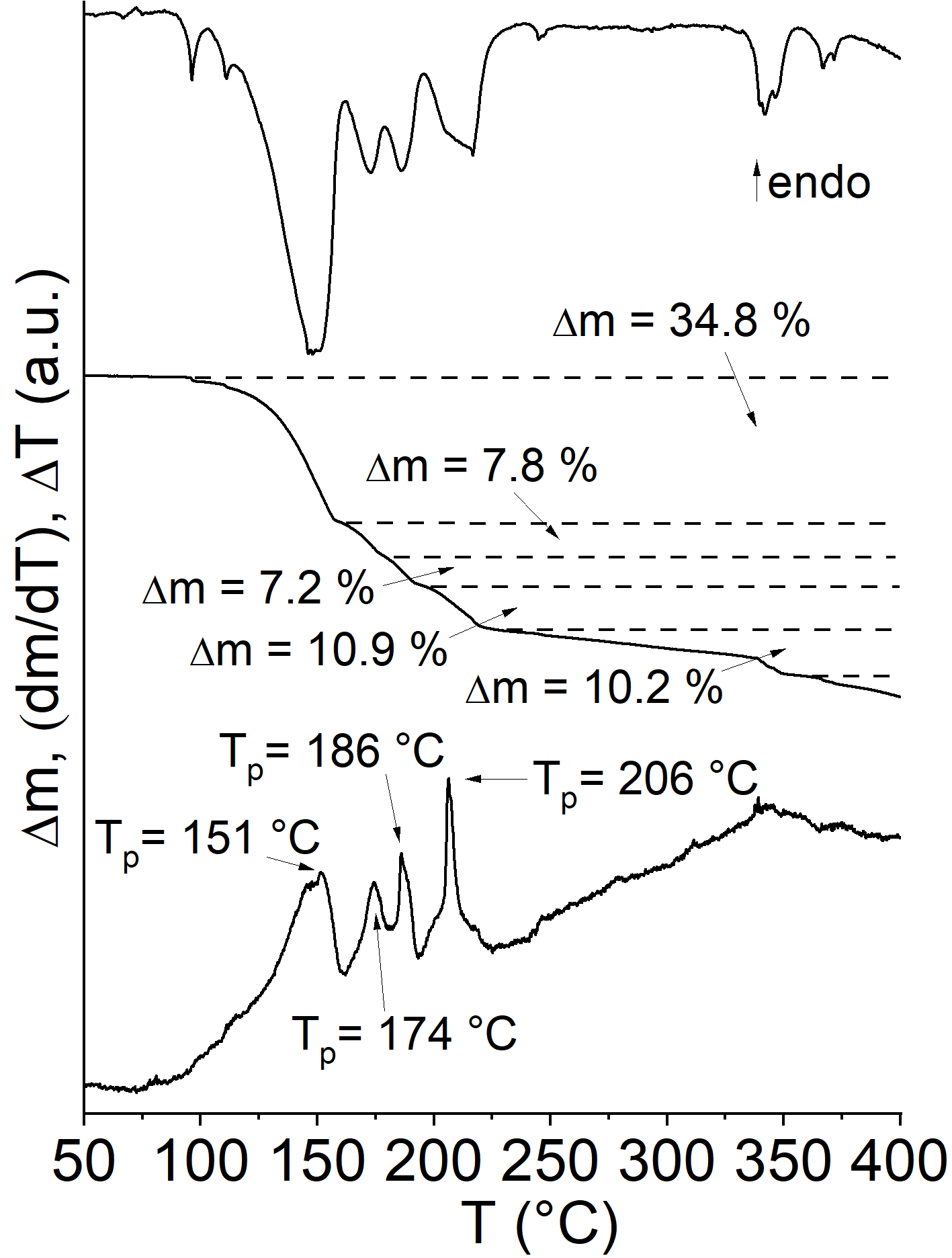

Supplement: Supplementary file 3 [file e-78-00755-sup6.png]

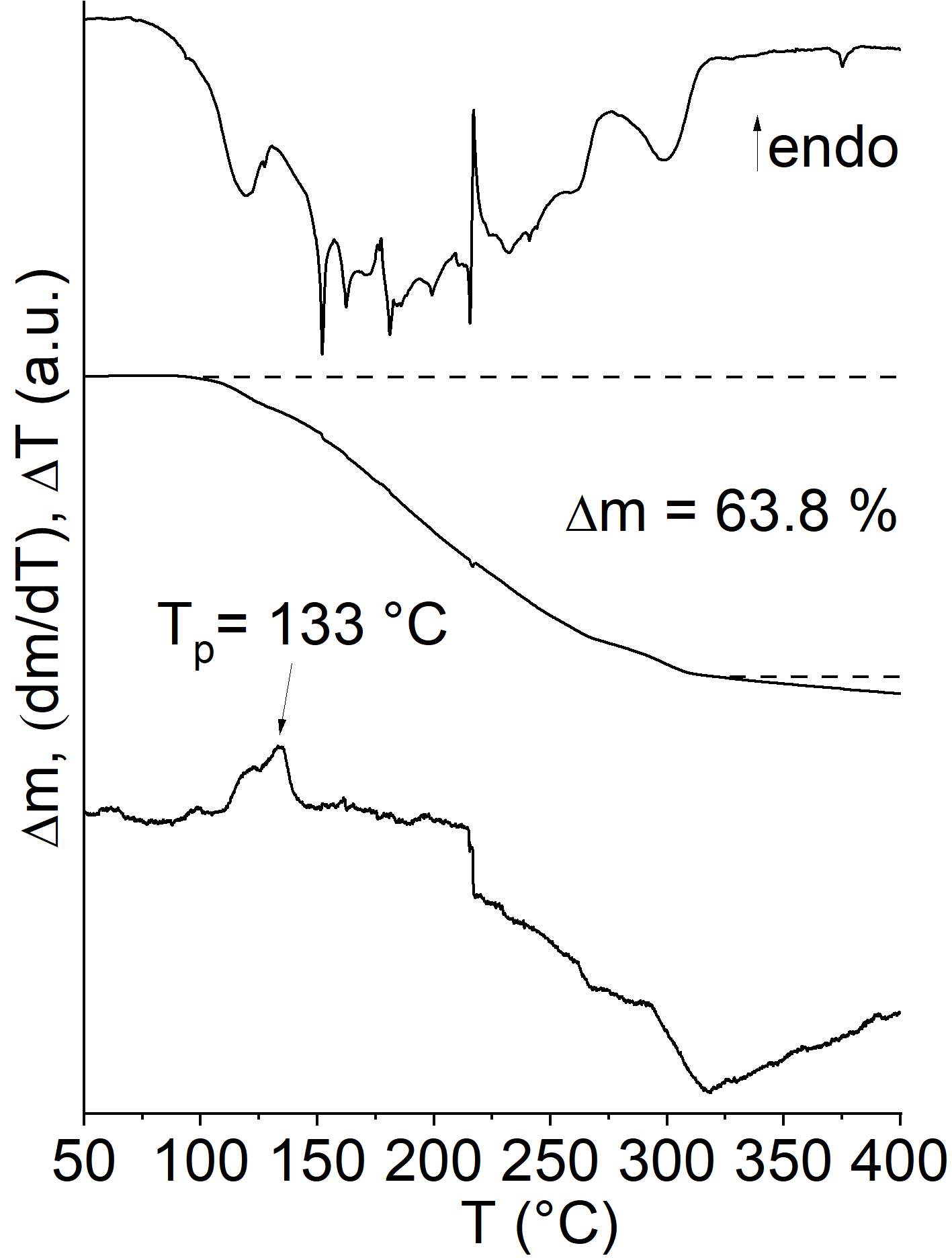

Supplement: Supplementary file 4 [file e-78-00755-sup7.png]

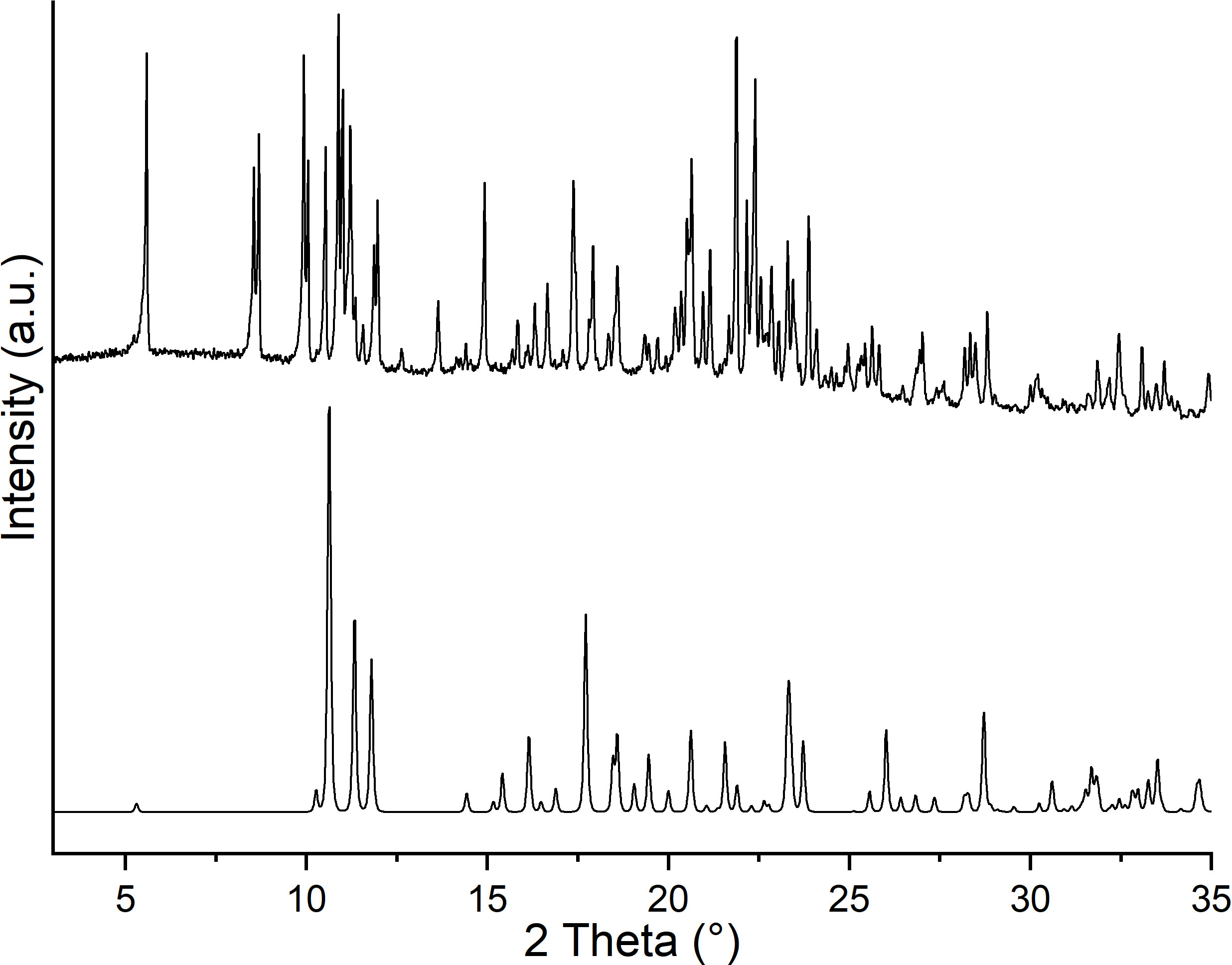

Supplement: Supplementary file 5 [file e-78-00755-sup8.jpg]
